# Isolation of *Synechocystis* Mutants Overproducing Mannitol Directly from CO_2_ via Laboratory Evolution under Increasing Salt Concentration

**DOI:** 10.1021/acssynbio.5c00344

**Published:** 2025-09-01

**Authors:** Wenyang Wu, Jente A. Jongbloets, Wei Du, Klaas J. Hellingwerf, Filipe Branco dos Santos

**Affiliations:** Molecular Microbial Physiology Group, Swammerdam Institute for Life Sciences, Faculty of Science, 1234 University of Amsterdam, Science Park 904, Amsterdam 1098 XH, The Netherlands

**Keywords:** mannitol, *Synechocystis*, adaptive laboratory evolution, genome resequencing, *pnp*

## Abstract

Mannitol is a naturally occurring C(6) polyol with a wide range of applications in the food and pharmaceutical industry. In a previous study, mannitol production was achieved via the direct conversion of CO_2_ in *Synechocystis* sp. PCC6803. However, a major barrier to future applications of this strain was its low production rate. In this study, three mutants were isolated after 164 generations of adaptive laboratory evolution under salt stress. These mannitol overproducing mutants were able to produce 27.71 mg L^–1^ OD_730_^–1^ mannitol under 350 mM salt stress when the OD_730_ reached 2, roughly 24 times higher than their parental strains. Whole-genome resequencing was then performed, revealing mutations in 2 genes—*pnp* and *sigA*/*rpoD1*—of the overproducing mutants when compared to the parental strain. Of these genes, *pnp* which encodes for polyribonucleotide nucleotidyltransferase was found to negatively affect mannitol production in cells via reverse engineering methods, in which the (partial) removal of *pnp* alone resulted in a 6.4-fold increase in the mannitol production. The work reported here substantially advances mannitol production capabilities in engineered *Synechocystis* sp. PCC6803 strains through adaptive evolution but also highlights the previously unrecognized negative regulatory role of *pnp* in mannitol synthesis.

## Graphical abstract

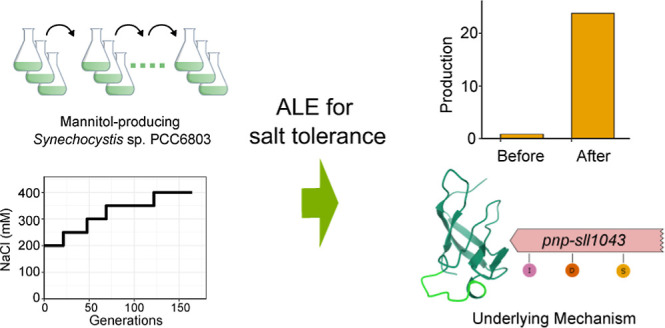

## Introduction

Sustainable chemical production is increasingly attracting public attention due to climate change, fossil fuel shortage, and energy security issues.^1,2^ Cyanobacteria are promising industrial microorganisms for sustainable chemical production because these organisms can directly fix atmospheric CO_2_ and convert it into specific chemical compounds via photosynthesis.^3–5^ This biosynthetic process by cyanobacteria not only decreases the level of atmospheric CO_2_ but also provides necessary chemical substances desired by human societies.^6^ Recently, the sustainable production of mannitol was achieved in cyanobacteria by heterologous expression of mannitol-1-phosphate-5-dehydrogenase (*mtlD*) and mannitol-1-phosphatase (*m1p*)^7–11^ or expression of the mannitol dehydrogenase to directly convert fructose into mannitol.^12^ In addition to low mannitol productivity, a major challenge in using cyanobacteria for mannitol production is the compound’s toxicity to the cells, which leads to instability in production even at low production rates.^8,9,11^ In a previous paper, we reported that mannitol production in a *Synechocystis* sp. PCC6803 (hereafter *Synechocystis*) increases salt tolerance and that making mannitol in cells under salt pressure can actually confer a selective advantage for these *Synechocystis* strains. This fitness benefit is even greater for mutants that lack the ability to produce endogenous compatible solutes (i.e., sucrose and glycosyl-glycerol) in response to salt stress conditions.^9^ Thus, a stable mannitol production system under salt stress conditions has been developed. However, although mannitol production in *Synechocystis* can be stabilized by salt stress, the consistently low productivity observed there still restricts its future application.

Adaptive laboratory evolution (ALE) is a method for strain engineering to improve tolerance against stress condition. During ALE, cells are repeatedly subcultured for prolonged periods under (increasing) stress conditions, ultimately leading to an increased growth rate.^13,14^ ALE can be used to improve growth rate and product yield, for adaption of strains to produce non-native products and increase the tolerance of strains toward a specific environmental stress.^15^ Combined with other biological techniques, the relationship between genomic change and adaptive phenotype can be exposed by genome resequencing.^16^ For mannitol-producing *Synechocystis* cells lacking the ability to produce native compatible osmolytes, we expect mannitol production to increase during ALE under increasing salt stress. We further expect such increase in production rate to be driven by the accumulation of one or more beneficial genetic mutations. By identifying and studying the mutations that appear in evolved populations, we hope to identify new mechanisms through which mannitol production can be further optimized in *Synechocystis*. The latter is an important step toward closing the gap between current production values and those required for viable practical applications.

In this study, the mannitol cassette (*mtlD* and *m1p*) was controlled by either the strong constitutive promoter P_trc1_ (ΔCS_M) or a native salt-inducible promoter P_ggpS_ (ΔCS_IM). Both were integrated in the background of the native compatible solute deficient *Synechocystis* strain (ΔCS). Our previous study demonstrated that the growth rate of ΔCS under 200 mM NaCl stress in BG-11 growth medium reached 70% of the maximum growth rate. The latter was observed under 50 μmol photons m^–2^ s^–1^ of incident red light^17^ with a 120 rpm shaking speed in an incubator at 30 °C, and mannitol productivity of ΔCS_M kept relatively stable under these conditions.^9^ Based on this information, we selected 200 mM NaCl as the initial stress condition for our ALE experiments. These experiments were further split under two conditions: (i) cells were exposed to either constant 200 mM NaCl stress or (ii) cells were exposed to increasing NaCl stress conditions. The latter condition can compensate for the cases in which evolved cells would be no longer sensitive to 200 mM salt.

Genomic sequencing of the obtained enhanced mannitol producers (EMPs) over ALE experiments revealed mutations in two genes to be related to an improvement in mannitol production. Among these, only the mutations located in *pnp* (polyribonucleotide nucleotidyltransferase) were discovered in all EMPs isolated. The product of *pnp* is part of degradosome in *Synechocystis*, which is involved in the degradation of mRNA.^18^ Its expression level has been reported to be upregulated in cells under heat shock.^19^ Its effect on mannitol expression in *Synechocystis* was further confirmed by the observation that the (partial) removal of *pnp* from the original mannitol producer (OMP) in the ΔCS_M background leads to increased mannitol production. This mutated OMP (OMP^#^1*) showed lower growth rate, more sensitivity to salt pressure, and a roughly 6.4 times higher mannitol production when OD_730_ reached 2. These results indicated that *pnp* indeed has an (in)­direct influence on mannitol production in *Synechocystis* but is most likely not the only gene affecting mannitol synthesis in EMPs, since the found increase of the OMP^#^1* strain does not fully account for the increase in mannitol found for the EMPs. This study demonstrates the successful use of adaptive laboratory evolution to enhance mannitol production in *Synechocystis*, highlighting its potential as a powerful strategy for strain improvement. Our findings provide new insights into the possible involvement of *pnp* in salt resistance in this cyanobacterium.

## Results

### Adaptive Laboratory Evolution of *Synechocystis* sp. PCC6803 under Salt Stress Conditions

In previous studies, we demonstrated mannitol production in *Synechocystis* through the heterologous expression of mannitol-1-phosphate-5-dehydrogenase (*mtlD*) and mannitol-1-phosphatase (*m1p*). We also found that the stability of mannitol production was highly improved under salt stress conditions in the background of native compatible solutes (sucrose and glycosyl-glycerol) deficient cells.^9^ The productivity we obtained at the time is far from what is required in industry though. In order to improve the production rate of mannitol in *Synechocystis*, we set out to apply ALE under salt stress conditions. The overall schematic of this process is shown in Figures 1 and 2. ΔCS (*ggpS* and *sps* double knockout mutant), ΔCS_IM (mannitol cassette driven by the salt-inducible P_ggpS_ promoter on the ΔCS background), and ΔCS_M (mannitol cassette driven by the P_trc1_ promoter on the ΔCS background) (see also Table S2) were evolved in three parallel experiments under either 200 mM of constant salt stress or under increasing salt stress (from 200 mM to 400 mM) based on growth–dilution cycles as described in the methods section.

**Figure 1:**
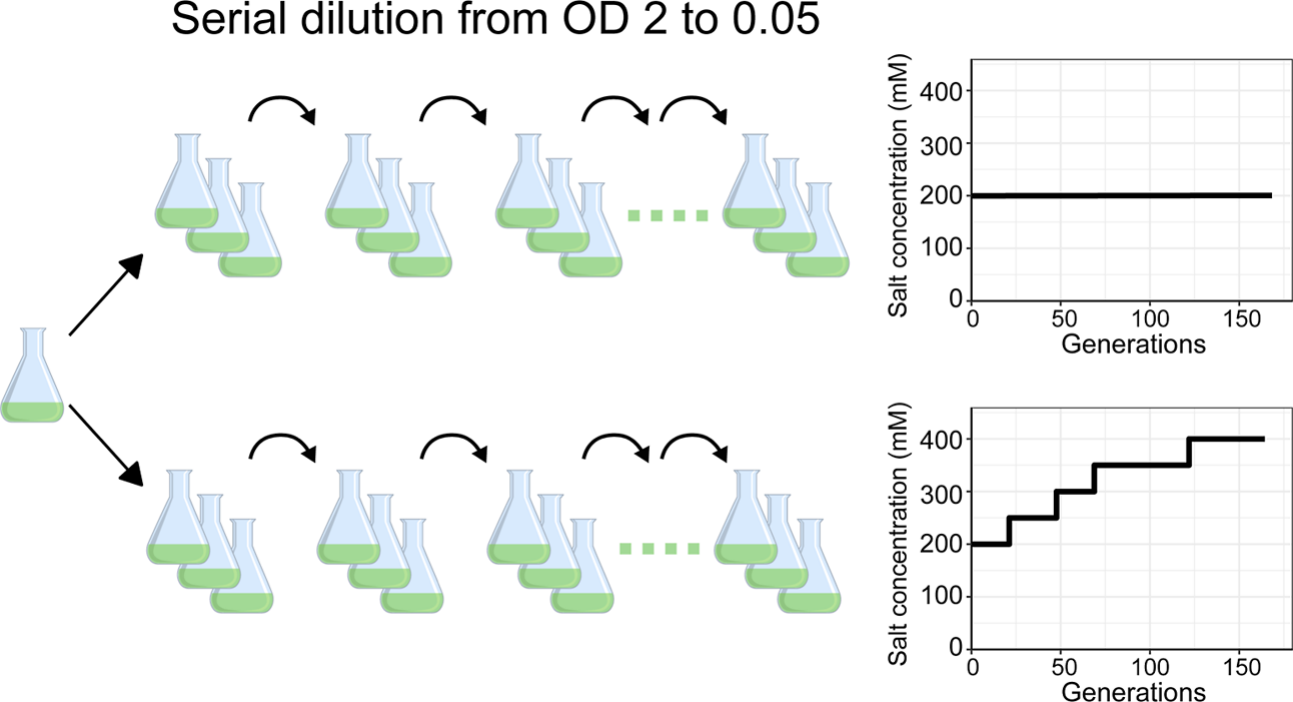
Experimental setup of the ALE process in this study. The whole ALE process of mutants ΔCS, ΔCS_IM, and ΔCS_M in three parallel runs under either constant 200 mM salt or increasing concentration of salt from 200 mM to 400 mM pressure. All the cultures were cultivated in flasks under previously mentioned conditions and cells were diluted and inoculated into fresh medium at an OD_730_ of 0.05 from an OD_730_ of ∼2.0 to maintain the exponential growth phase.

**Figure 2:**
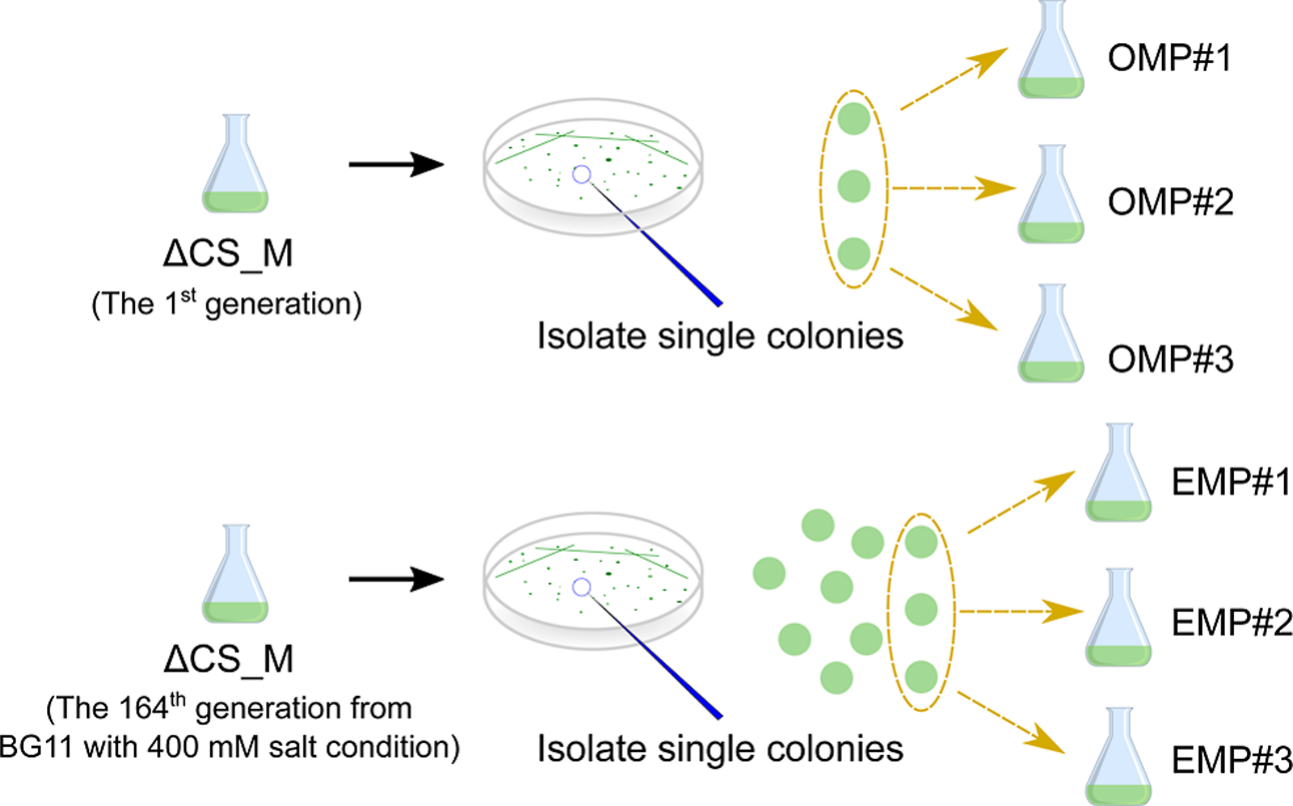
Solid BG-11 plates were used to isolate single colonies from ΔCS_M either at the 1st generation or the 164th generation. Three of ten single colonies selected from ΔCS_M at the 164th generation under 400 mM salt pressure which showed higher extracellular mannitol production were identified as enhanced mannitol producers EMP^#^1, EMP^#^2, and EMP^#^3, respectively. Three random single colonies from ΔCS_M at the 1st generation were identified as original mannitol producers OMP^#^1, OMP^#^2, and OMP^#^3, respectively.

All cells were repeatedly transferred to fresh media during the exponential growth phase. The growth rate of ΔCS, ΔCS_IM, and ΔCS_M under 200 mM constant salt stress increased rapidly at the beginning of ALE after which the growth rate stabilized to 0.052 h^–1^ around the 30th generation (Figure 3A). The extracellular mannitol production of ΔCS_IM and ΔCS_M increased 1.64- and 2.15-fold (Figures 3A and S1), respectively, after 100 generations of ALE, resulting in 0.25 and 1.23 mg L^–1^ OD_730_^–1^ of mannitol. The huge differences in mannitol productivity between ΔCS_IM and ΔCS_M are most likely caused by the difference in the expression level of the promoters, since P_trc1_ is regarded as one of the strongest promoters in *Synechocystis*, while the expression level of the native promoter of *ggpS*—even under salt stress—is low in comparison to P_trc1_.^20^

**Figure 3:**
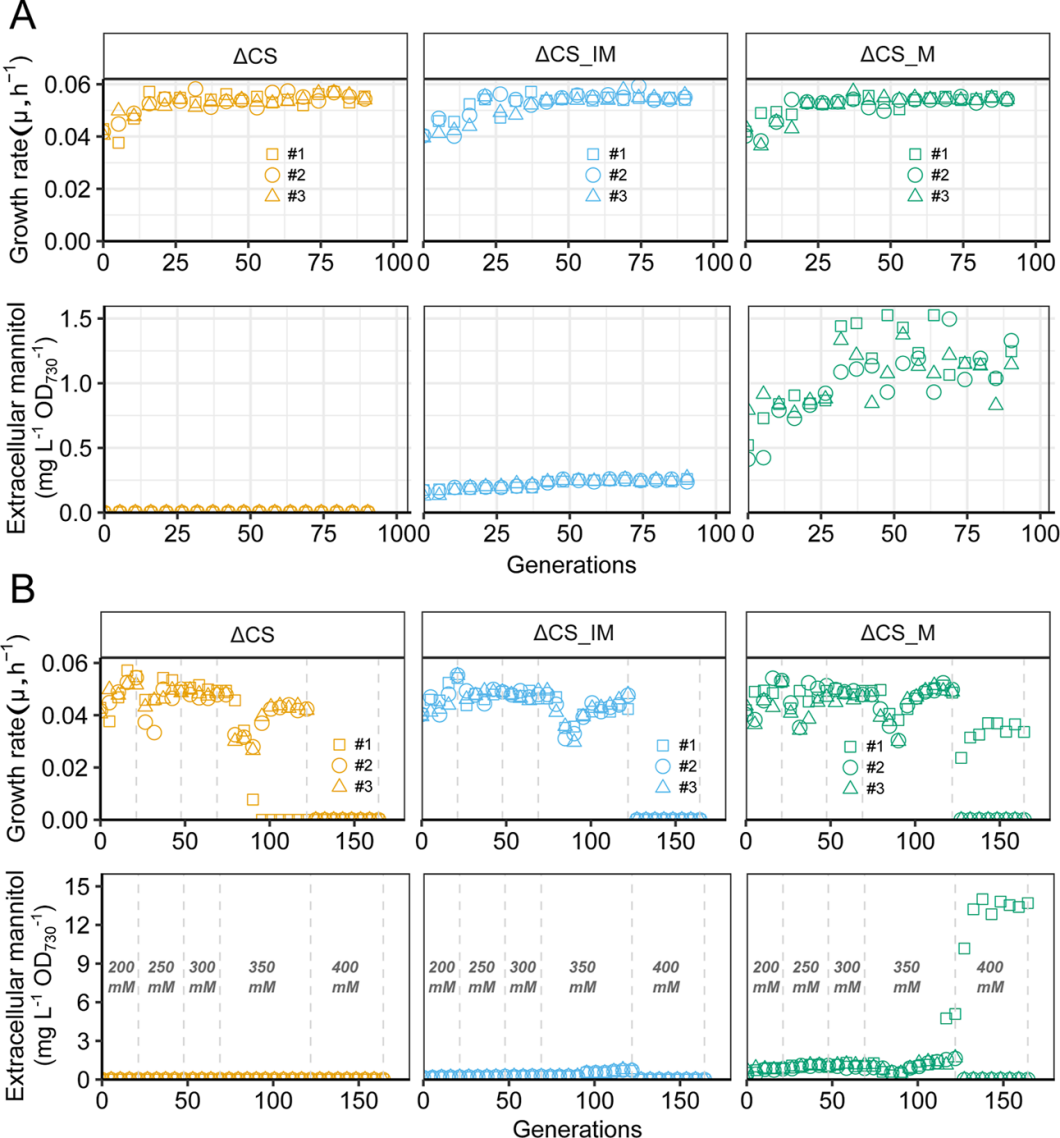
Growth rate and extracellular mannitol production of ΔCS (*ggpS* and *sps* double knockout mutant), ΔCS_IM (mannitol cassette under the salt-inducible P_ggpS_ promoter on the ΔCS background), and ΔCS_M (mannitol cassette under P_trc1_ promoter on the ΔCS background) during the process of ALE either under the constant 200 mM salt pressure (A) or increasing salt pressure (B). The extracellular mannitol production was determined when the OD_730_ reached 2. Growth rate was determined by measuring OD_730_ every 2 h for a minimum of 10 h at the next day of dilution. The numbers in the plots indicate the salt concentrations (in mM) used during ALE.

For the evolution process under increasing salt concentrations, we used the simple rule to increase salt concentrations by 50 mM, once the observed growth rate was equal to or larger than the growth rate obtained at the end of the previous conditions. Figure 1 illustrates this simplified process of the ALE experiment as a gradual increase of salt concentrations. We found that after around 21 generations in the 200 mM salt condition, the growth rate of ΔCS, ΔCS_IM, and ΔCS_M had increased significantly and then stabilized. The salt concentration was then increased to 250 mM. This brought a higher osmotic burden to the cells, and understandably, an abrupt decrease in growth rate was observed. Eventually, the pressure of 250 mM salt was alleviated, and the growth rate stabilized to ∼0.049 h^–1^. Interestingly, after increasing the salt concentration to 300 mM, we found that cells adapted to 250 mM salt can easily adapt to 300 mM salt concentration because the growth rate did not show any change. Further increase of the salt concentration to 350 mM led now to a large decrease in growth rate and the loss of one replicate culture from the ΔCS as it stopped growing after a few rounds of growth–dilution cycles, while the growth rate of the other cultures recovered after ∼20 generations. Surprisingly, when 400 mM salt concentration was used, only one culture (one of the ΔCS_M replicates) was able to survive and stabilized to a growth rate lower than any growth rate seen in previous conditions, just 0.033 h^–1^ at the 164th generation (Figure 3B). The extracellular mannitol production of ΔCS_IM and ΔCS_M, on the other hand, increased with the increasing salt conditions and reached 0.72 mg L^–1^ OD_730_^–1^ and 2.80 mg L^–1^ OD_730_^–1^, respectively, at the 121th generation in the 350 mM salt condition. The ΔCS_M culture growing in 400 mM salt was able to produce 13.69 mg L^–1^ OD_730_^–1^ at the 164th generation (Figure 3B and Figure S2). Overall, the ALE strains showed a significant increase in extracellular mannitol under increasing salt stress with 4.5-fold and 24-fold increases observed for ΔCS_IM and ΔCS_M, respectively (Figure 4).

**Figure 4:**
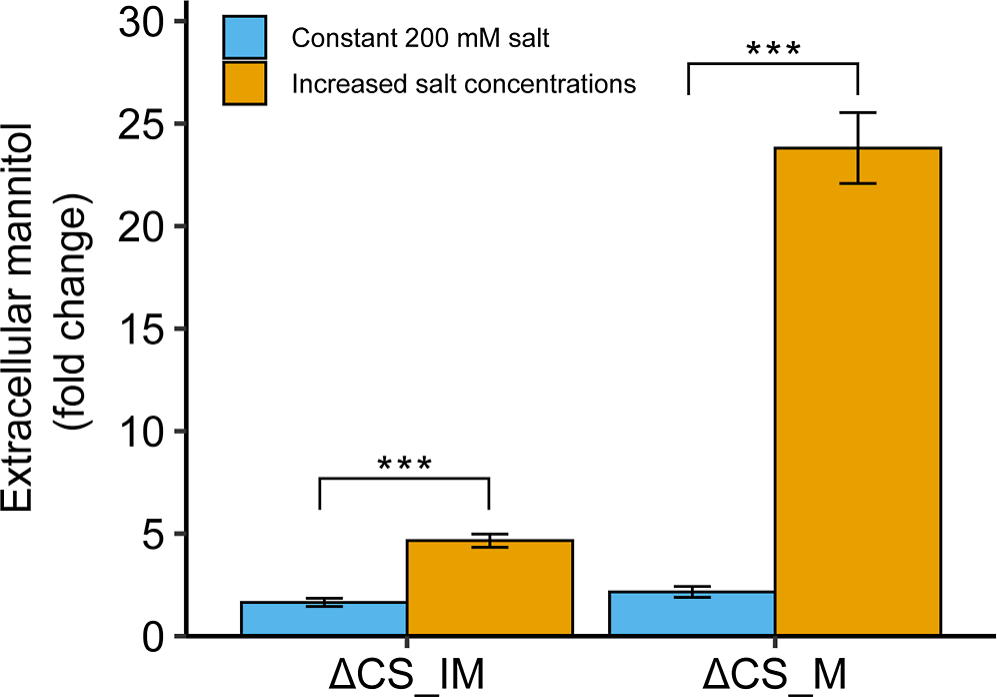
Improvement in mannitol production of ΔCS_IM and ΔCS_M after ALE. Mannitol was determined when the OD_730_ reached 2. Error bars represent the standard deviation of three biological replicates. Asterisks indicate statistical significance (*P* < 0.01) as determined by a two-tailed *t*-test.

Overall, increasing extracellular mannitol production was observed during the whole evolution process (Figures 3 and S1 and S2), indicating that adaptive evolution under salt stress successfully led to higher mannitol production. Both conditions of constant and increasing salt stress can result in cells producing mannitol at higher rates, with the increasing salt stress condition leading to the largest increase in mannitol production. Our results show that the evolutionary benefit obtained from mannitol production under salt stress is tightly related to the perceived intensity of the stress. Excess mannitol production does not appear to provide additional evolutionary benefits, and further increases in production are observed only following an increase in salt concentration. This finding supports the role of salt stress as a stabilizing factor in mannitol production.

### Identification of Enhanced Mannitol Producers (EMPs) from ALE

To isolate enhanced mannitol producers (EMPs) from the process of ALE, we plated the evolved ΔCS_M populations at the 164th generation on solid BG-11 plates with 200 mM salt (Figure 2). We then picked ten random single colonies and cultured them individually in BG-11 to assess their mannitol production rate. Out of the 10 colonies, we picked the three isolates with the highest extracellular mannitol production when the OD_730_ reached 2 (data not shown). These isolates were identified as enhanced mannitol producers (EMPs) and named EMP^#^1, EMP^#^2, and EMP^#^3. In order to compare mannitol production of the EMPs with the parental ΔCS_M, we used the same isolation method as for the EMPs and picked three random single colonies and named these original mannitol producers (OMPs) as OMP^#^1, OMP^#^2, and OMP^#^3.

As shown in Figure 5A (also in Figure S3), the EMP isolates grew significantly more slowly than the OMP isolates under normal cultivation conditions without salt stress. When grown in the presence of salt conditions, the growth of the EMPs was relatively similar to the growth observed without salt stress, while the growth of the OMPs decreased to a significantly lower growth rate. Furthermore, drop experiments revealed that only the EMP isolates were able to survive 350 mM and 400 mM salt (Figure 5A,D), demonstrating the improved salt tolerance of the evolved strains. Figure 5B (also in Figure S4) shows that the mannitol production (at an OD_730_ of 2) of the EMPs increased significantly, ∼24 times more than that measured in the OMP isolates. The highest total and extracellular mannitol production observed in EMPs was 31 and 22 mg L^–1^ OD_730_^–1^, respectively, under 350 mM salt stress, whereas the highest total and extracellular mannitol production in OMPs was only 3 and 2 mg L^–1^ OD_730_^–1^ (Figure 5B). In addition, we observed that without salt stress, mannitol production in both the OMPs and EMPs was the lowest of all salt conditions after 14 days of cultivation (Figures 5C and S5 and S6). This illustrates the tight pressure under which mannitol production in *Synechocystis* is, where overproduction of mannitol is clearly an evolutionary burden. Furthermore, we found in our previous study that biosynthesis of mannitol in *Synechocystis* without salt pressure leads to rapid accumulation of mutations on the mannitol cassette resulting in loss of mannitol production. The observation in this study of reduced mannitol production in both OMPs and EMPs without salt suggests this process is likely to be also occurring in these strains. In conclusion, EMPs produced approximately 700 mg L^–1^ of mannitol after 14 days of cultivation under salt concentrations above 350 mM, representing the highest total mannitol concentration achieved in this study. In contrast, the OMPs produced only around 45 mg L^–1^ under the same conditions and duration (Figures 5C and S6).

**Figure 5:**
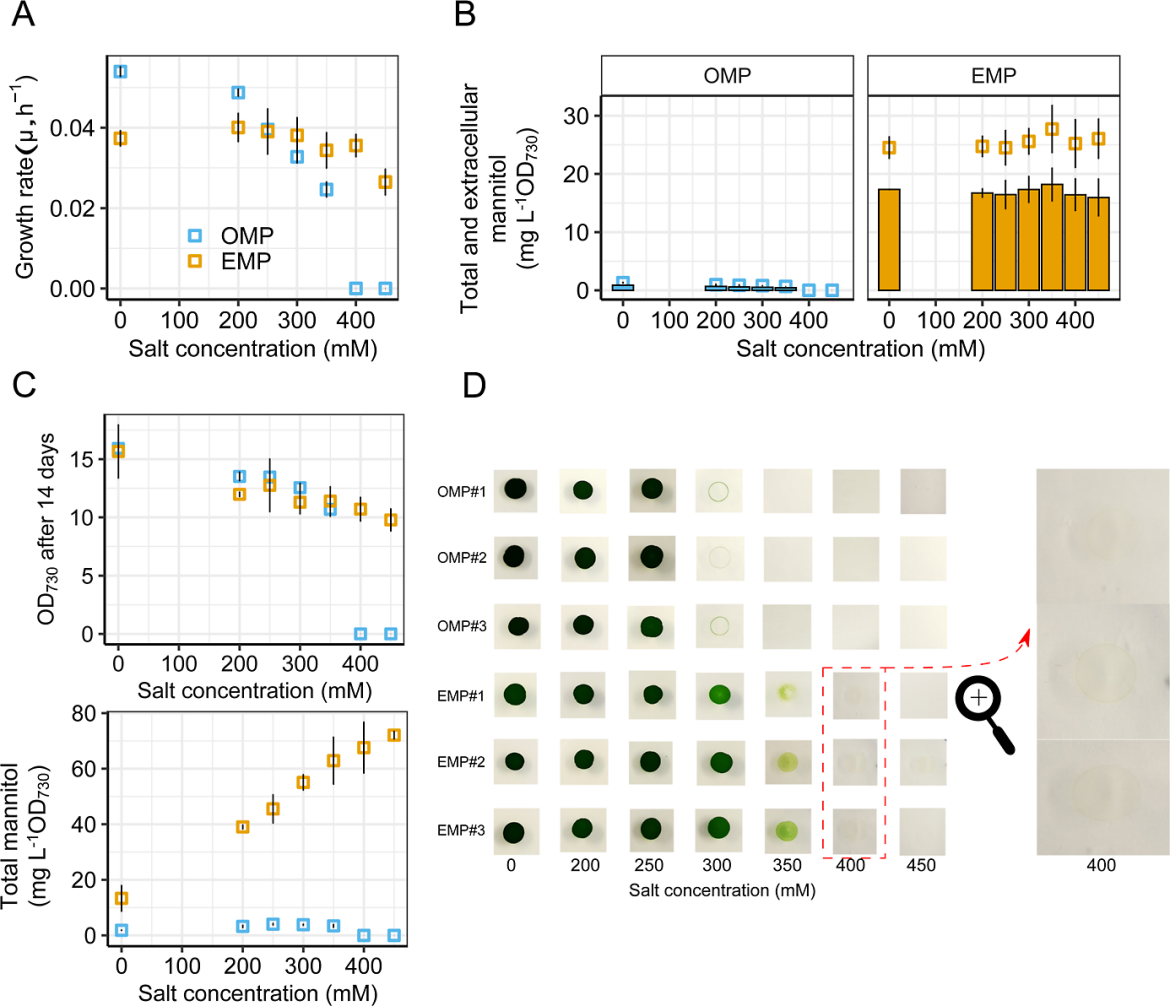
Comparison of OMP and EMP phenotypes under varying salt concentrations (0 mM, 200 mM, 250 mM, 300 mM, 350 mM, 400 mM, and 450 mM). (A) Growth rate of the OMP and EMP isolates. (B) Determination of total and extracellular mannitol production of the OMP and EMP isolates when the OD_730_ reached 2. Bars: extracellular mannitol production; empty square: total mannitol production. (C) Determination of OD_730_ and total mannitol production of the OMP and EMP isolates after 14 days continuous cultivation. (D) Determination of salt resistance ability of different OMP and EMP isolates (#1, #2, and #3) on solid BG-11 plates with 0 mM, 200 mM, 250 mM, 300 mM, 350 mM, 400 mM, and 450 mM salt. The strains OMP^#^1, OMP^#^2, OMP^#^3, EMP^#^1, EMP^#^2, and EMP^#^3 were grown to OD_730_ = 1.0. The cell density was determined by the CASY counter and diluted as 12,500 cells μL^–1^ and then 5 μL of OMP^#^1, OMP^#^2, OMP^#^3, EMP^#^1, EMP^#^2, and EMP^#^3 were transferred to solid BG-11 plates with 0 mM, 200 mM, 250 mM, 300 mM, 350 mM, 400 mM, and 450 mM salt. The plates were incubated for 10 days at 30 °C. Error bars represent the standard deviation from three biological replicates, each derived from a distinct isolate.

### Whole-Genome Resequencing of the Evolved Strains

The large increase in mannitol production between OMPs and EMPs motivated us to look deeper into the underlying genetic changes that are driving the increase in mannitol production seen in our ALE experiments. We used whole-genome sequencing technology to identify potentially interesting mutations and confirmed them independently using Sanger sequencing.

The genomes of three isolates from the parental ΔCS_M (OMP^#^1, OMP^#^2, and OMP^#^3) and three isolates from the evolved ΔCS_M (EMP^#^1, EMP^#^2, and EMP^#^3) were sequenced. We used breseq^21^ to align and compare the obtained reads to a custom-made reference based on assembly ASM27026v1 obtained from NCBI. Since *Synechocystis* is known to be polyploid,^22^ mutations do not necessarily need to be fully segregated in order to remain within the population. To include such mutations in our analysis, we used breseq in polymorphism mode. This resulted in the detection of 1001 unique mutations in 85 different genes. To reduce this number and remove false positives, we further analyzed the found mutations using the percentage of reads supporting each mutation in each sample. We used Principal Component Analysis (PCA) and enrichment analysis to select potential mutations. The first two Principal Components (PC) found by PCA were able to discriminate between the generations (PC 1, explaining 46% of all variation) and between evolved samples (PC 2, explaining 32% of variation), as shown in Supporting Information Figure S7. We selected the 10 most (in absolute sense) contributing mutations from each PC for further investigation. In our enrichment analysis, we focused on genes with mutations that increased in abundance between the parental and evolved isolates (Figure S8).

After combining the results of our PC and enrichment analysis, four mutations (Figure 6A) sparked our interest: one deletion (D), two substitutions (S), and one insertion (I). Of the four mutations, three (1 D, 1 S, and 1 I) were located in *pnp* (polyribonucleotide nucleotidyltransferase). The other mutation (a substitution) was found in a gene annotated as RNA polymerase sigma factor *SigA (sigA/rpoD1)*.

**Figure 6:**
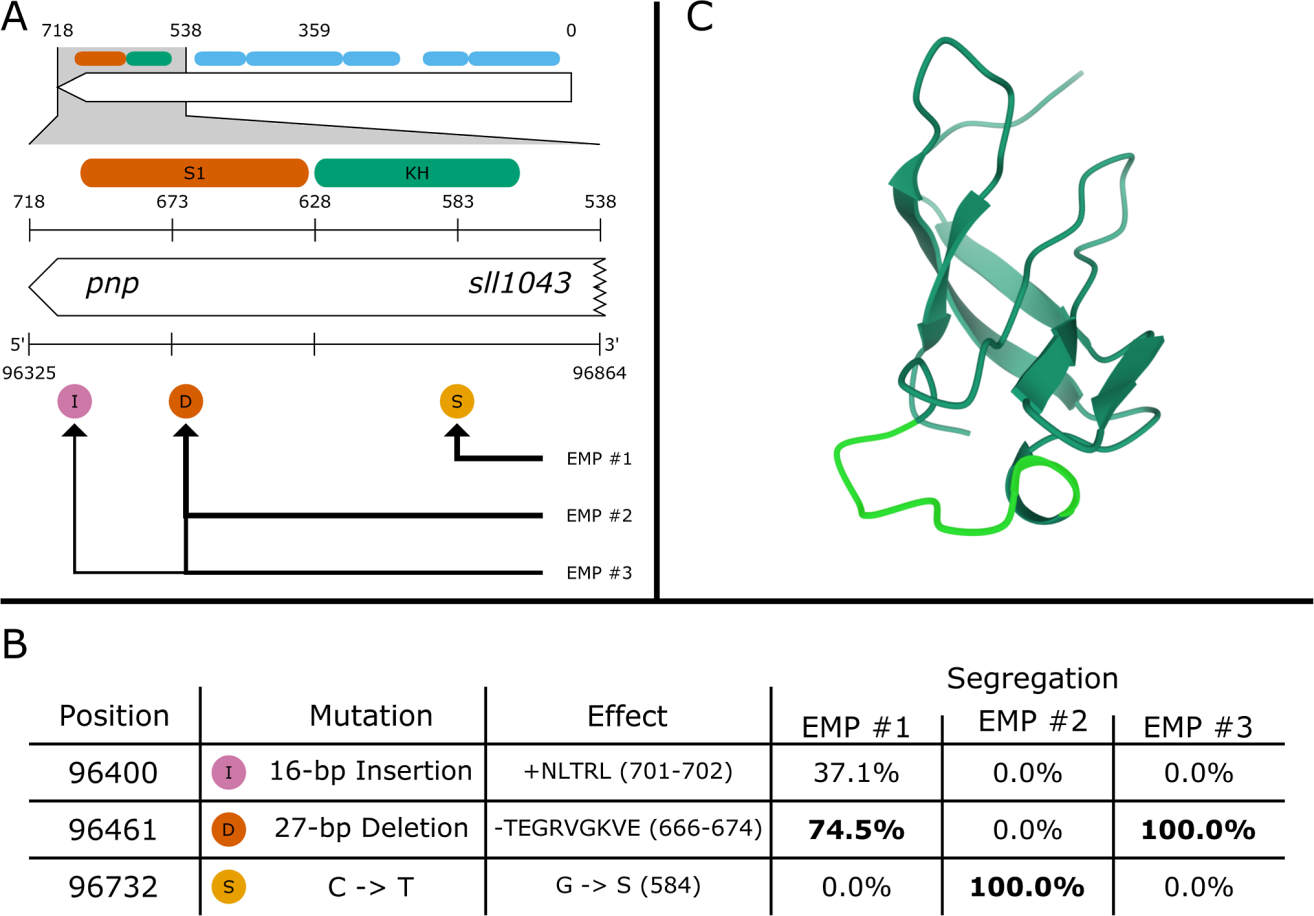
Identified mutations in *pnp* (polyribonucleotide nucleotidyltransferase). (A) 3 mutations were detected in *pnp* in a region annotated as the S1 and KH domain. (B) We detected a 16-bp insertion (I), a 27-bp deletion (D), and a G584S substitution (S). Of these mutations, we were able to confirm 2 by Sanger sequencing (in bold). (C) 27-bp deletion highlighted (in light green) in the 3D resolved S1 domain structure. The structure was obtained from the Protein Data Bank under accession ID 1SRO.

Interestingly, the same 27-base deletion was found in both EMP^#^1 and EMP^#^3 (based on 74.5% and 100% of the reads, respectively). Additionally, EMP^#^1 seemed to contain an insertion (a duplicated repeat) in 37.1% of the reads, which was relatively close to the deletion. EMP^#^2, on the other hand, clearly exhibited a different genotype. Here, breseq detected a single point mutation (G584S) in *pnp* (changing a glycine into a serine at position 584 in the amino acid sequence) and a R188Q SNP in s*igA/rpoD1* (changing an arginine to a glutamine at position 188), both supported by 100% of reads from EMP^#^2.

Using Sanger sequencing, we were able to confirm 3 out of the 4 mutations detected by breseq (bold frequencies in Figure 6B). The only mutation we were not able to confirm was the inserted repeat detected in EMP^#^1, which may have been due to the low level of segregation of this mutation in the isolate.

Using domain annotations of *pnp* (UniProt ID: P72659) from InterPro,^23^ we placed the G584S substitution in a region annotated as a KH Type-1 domain (Figure 6A). This mutation changes the main motif of the KH domain^24^ from *VIG__G__I* to *VIG__S__I*. The 27-bp deletion falls within a region annotated as the S1 domain. The deletion removes a linker section just before and at the beginning of the fourth β-strand (Figure 6C). The KH and S1 domains together are thought to be responsible for binding the RNA^24,25^ so that it can be degraded by *pnp*.^26^ Similar mutations in the main motif of the KH domain (i.e., substitutions of the first glycine) have been shown to lead to significantly less RNA degradation by *pnp* due to less stable binding of the substrate to the domain.^27,28^ The substitution R188Q found in *sigA/rpoD1* (UniProt ID: P74565) falls just outside of the annotated sigma-70 region 2 domain. *rpoDI* has been reported as a RNA polymerase sigma factor (σ_70_) in *Synechocystis*, and its expression is highly increased in salt stress.^29^

The finding of mutations in *pnp* and *sigA/rpoD1* both associated with modulating gene expression in response to stress conditions is not surprising as salt pressure was exerted throughout the whole ALE experiment. With *pnp* being related to the degradation of mRNA and *rpoD1* as a transcription initiation factor, the mutations found suggest that *Synechocystis* was able to increase mannitol production by altering gene regulation. We decided to further investigate the mutations found in *pnp*, as all evolved strains contained highly segregated mutations in this gene.

### Reverse Engineering of *pnp* in OMP

To investigate whether *pnp* is driving the observed increase in mannitol synthesis, we attempted to remove *pnp* from the OMP^#^1 (Figure S9). Growth profiles were performed with this strain (OMP^#^1*) using different salt concentrations (0, 200, 250, 300, 350, 400, and 450 mM). We found OMP^#^1* had a ∼30% decreased growth rate as compared to its parental strain (OMP^#^1) at 0–300 mM of salt (Figure 7A). Drop experiments revealed that *pnp* (partial) deletion reduced salt resistance as the OMP^#^1* was only able to tolerate up to 250 mM salt stress on the solid BG-11 plate (Figure S10). However, while both growth rates and salt resistances were negatively affected by the removal of *pnp*, mannitol production in flasks under various salt stress (0 mM, 200 mM, 250 mM, and 300 mM) was still elevated, roughly 6.5 or 2 times, respectively, either when the OD_730_ reached 2 or after 14 days of cultivation (Figures 7B–D and S11 and S12). This indicates that *pnp* indeed has an impact on mannitol production; however, this increase is still small in comparison to the mannitol production found in the EMPs (∼3.5 times lower). This difference is likely to be caused by the fact that the mutations that were accumulated during the ALE experiment are different from the knockdown of *pnp* abundance that was tested. Nonetheless, it clearly shows *pnp*’s involvement in the mannitol producing phenotype selected, which in combination with other mutations leads to the also observed restoration of growth rate and increased salt tolerance in the evolved *Synechocystis*.

**Figure 7:**
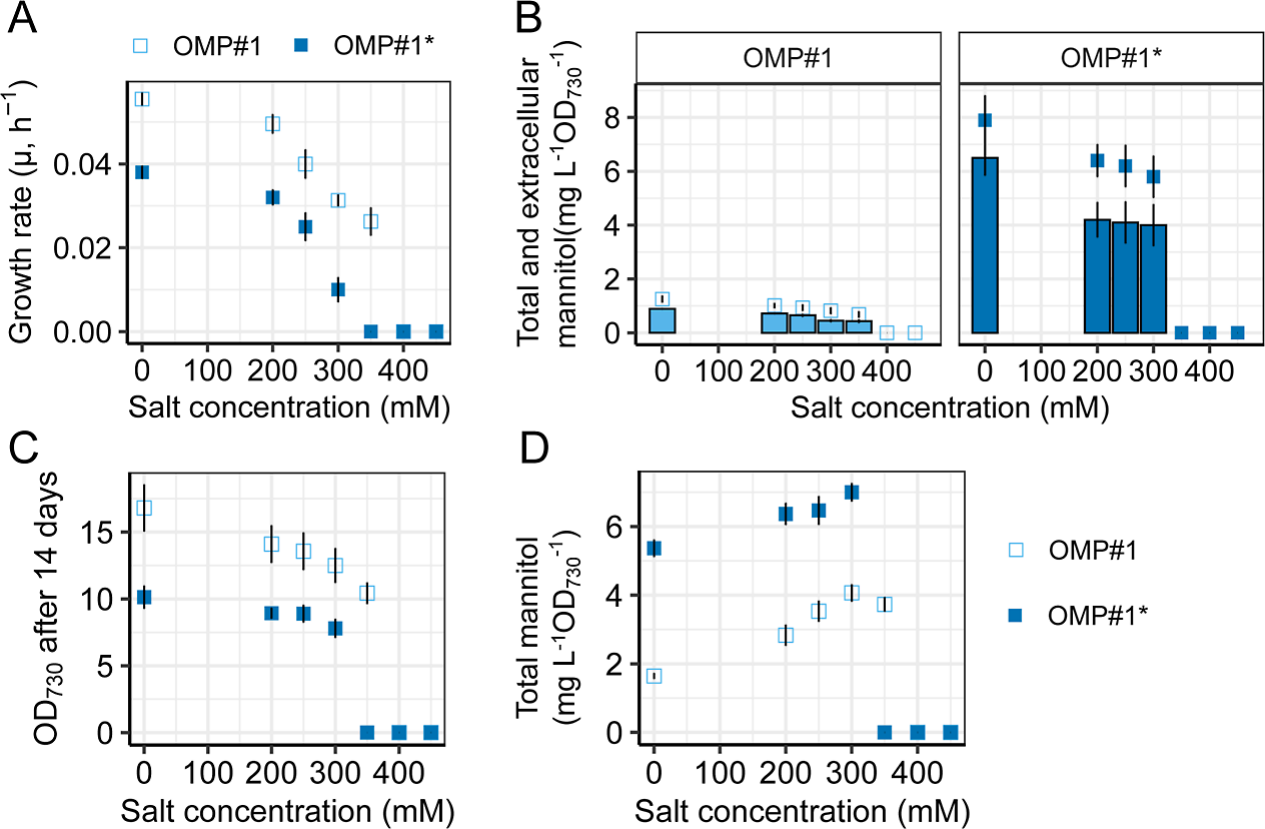
Phenotype comparisons between OMP^#^1 and OMP^#^1*. (A) Growth rate of OMP^#^1 and OMP^#^1* under different salt concentrations (0 mM, 200 mM, 250 mM, 300 mM, 350 mM, 400 mM, and 450 mM). (B) Determination of total and extracellular mannitol production of OMP^#^1 and OMP^#^1* when the OD_730_ reached 2 under different salt concentrations (0 mM, 200 mM, 250 mM, 300 mM, 350 mM, 400 mM, and 450 mM). Bars: extracellular mannitol production; empty circles: total mannitol production. (C) Determination of OD_730_ and (D) total mannitol production of OMP^#^1 and OMP^#^1* after 14 days of continuous cultivation under different salt concentrations (0 mM, 200 mM, 250 mM, 300 mM, 350 mM, 400 mM, and 450 mM). Error bars represent the standard deviation of three biological replicates.

## Discussion

In this study, evolved strains of *Synechocystis* were obtained using ALE in BG-11 medium supplemented with different levels of salt. Using this method, we were able to obtain strains with enhanced mannitol production traits, roughly 24 times higher than that of their parental strains. In addition, adaptive evolution under salt stress not only increased mannitol production but also increased salt tolerance from 300 mM to 450 mM. Genome resequencing analysis revealed that the only gene mutated in all the enhanced mannitol producers (EMP^#^1, EMP^#^2 and EMP^#^3) was *pnp*. Reverse engineering approaches based on its partial removal revealed that *pnp* has indeed a large effect on salt resistance and mannitol synthesis.

We showed that the carbon flux in *Synechocystis* was partially redirected to mannitol under salt stress. Tailoring of carbon partitioning to enhance the production rate in engineered strains is a common method.^30^ Already in 2014, mannitol production was improved in *Synechococcus* sp. PCC7002 by knocking out *glgA1* and *glgA2*. This strategy blocks part of the glycogen synthesis and more carbon becomes available to synthesize mannitol.^8^ A similar strategy was adopted for increasing lactic acid in *Synechocystis* by removing an endogenous carbon sink, such as glycogen, via the deletion of *glgC*.^31^ These papers inspire us to further enhance mannitol production in EMPs in the future by redirecting carbon flux into mannitol synthesis via changes in the metabolic network, for example, by knocking out *glgA1* and/or *glgA2* to block glycogen synthesis. A previous study also demonstrated that impaired glycogen synthesis results in increased salt sensitivity in *Synechocystis*.^32^ We speculate that introducing mannitol production into a *glgA1* and/or *glgA2* deletion mutant could yield results similar to those described here. However, strains lacking glycogen synthesis may experience growth impairments and difficulties in coping with diurnal cycles.

With genomic sequencing, we detected four mutations in two genes, *pnp* and *rpoDI*, which could explain the increased mannitol production observed in the EMPs after ALE. Of these two genes, all isolates contained mutations in *pnp* and we decided to focus our investigation on this locus. The reverse engineering experiments revealed that removing *pnp* does improve mannitol production in the parental mannitol producer (OMP^#^1); however, (partial) removal of *pnp* also reduces the growth rate and increases salt sensitivity. A limitation of this reverse engineering study is the absence of a complementation experiment in which *pnp* would be reintroduced into the *pnp* (partial deletion) strain. Future experiments are needed to complement *pnp* in this background to more definitively confirm its role in mannitol production. Nevertheless, based on the current data, we speculate that mutations found in *pnp* are present to adjust the salt response. A previous study found the expression level of *pnp* to be upregulated in high temperature,^19^ whereas its specific mechanisms on osmotic pressure regulation in *Synechocystis* are still unclear. In this study, we found that removing *pnp* from the chromosome of OMP#1 via kanamycin-based segregation was challenging. This observation is supported by the finding that *pnp* is responsible for RNA polyadenylation in *Synechocystis* and inactivation of *pnp* is lethal.^33^ Thus, we hypothesize that *pnp* may modulate the expression of key metabolic pathways, such as glycogen synthesis and photosynthesis, in *Synechocystis*. The mutations identified in *pnp* may indirectly enhance mannitol production by influencing these interconnected metabolic processes. Although the mannitol production of OMP^#^1* was increased roughly 6.5 times, the partial *pnp* deletion on its own cannot fully explain the extremely high mannitol production found in the EMPs, which is roughly 24 times higher than that in the OMPs under the same conditions. From this finding, we conclude the EMP phenotype is most likely obtained by altering the way *pnp* regulates gene expression, possibly in combination with a more diverse group of other mutations which were not taken along in our reverse engineering efforts. For instance, in EMP^#^2, *rpoDI* appeared to be mutated as well. The latter seems a likely candidate for playing an important role in the regulation of mannitol production in salt stress conditions. *RpoDI*, a sigma factor of RNA polymerase, is required for initiation of transcription^34^ and its expression level is known to be enhanced by salt stress.^29^

To establish a better regulation of mannitol production in response to salt stress, the native promoter of *ggpS* in *Synechocystis* was used to drive the expression of the mannitol cassette. *GgpS* in *Synechocystis*, encoding glucosyl-glycerol phosphate synthase, which is involved in glucosyl-glycerol synthesis has been identified as a salt-dependent gene.^35^ So, its native promoter is regarded as a salt-inducible promoter. Using ALE, we were able to increase mannitol production under control of this promoter roughly 1.6 times under either constant or 4.7 times the increasing salt pressure. However, compared to the productivity found in the EMPs, the evolved ΔCS_IM produces 70 and 25 times less extracellular mannitol, respectively, translating to 0.26 mg L^–1^ OD_730_^–1^ after 90 generations under 200 mM salt stress and 0.72 mg L^–1^ OD_730_^–1^ after 122 generations up to 350 mM salt stress.

In this study, we aimed to develop strains with mannitol productivity levels of interest for potential future applications. This means that the evolved ΔCS_IM does not meet our selection criteria. The evolved ΔCS_IM, however, does have the advantage of potentially being a very stable mannitol producer since the use of a salt-inducible promoter allows the strain to adjust mannitol productivity based on the experienced salt concentration in the environment. This trait would prevent mannitol from being oversynthesized in normal BG-11, which we know leads to rapid loss of mannitol productivity.^9^ We believe the lower mannitol production observed in ΔCS_IM is due to the relatively weak activity of its promoter. To address this issue, we propose to use a stronger salt-inducible promoter, such as *CrGPDH3*, which was identified as a very strong salt-inducible promoter in the microalgae *Chlamydomonas reinhardtii*.^36^ The effectiveness of this promoter in *Synechocystis* is still debated and requires further research. It is also worthwhile to test if the use of inducible promotors indeed increases the stability of mannitol production in conditions without salt stress.

## Conclusions

In this study, we demonstrate the use of adaptive laboratory evolution (ALE) not only to obtain an evolved strain with improved mannitol production but also to explore the factors contributing to this enhancement. The most promising strain generated using this approach was able to produce 24 times more mannitol compared with its parental strain. The mutations on *pnp* in the genome of EMPs compared with OMPs were identified by genome resequencing and the function of *pnp* was further studied by a reverse engineering strategy. The partial *pnp* deletion strain led to an increase of roughly 6.5 times more mannitol production, compared with its parental strain, which demonstrates that *pnp* influences mannitol production in *Synechocystis*. The performed experiments demonstrate the power of using ALE for rapidly improving mannitol production in *Synechocystis* and as a relevant biotechnological tool for the optimization of cell factories.

## Methods and Materials

### Bacterial Growth Conditions

The laboratory-evolved and engineered *Synechocystis* were grown on solid BG-11 plates with 1.5% agar or liquid BG-11 medium with 50 mM PIPPS [piperazine-N,N′- bis­(3-propanesulfonic acid)] buffer (pH 8) under a red-light intensity of ∼50 μmol photons m^–2^ s^–1^ in a shaking incubator of 120 rpm at 30 °C.^9^ Antibiotics were added to liquid BG-11 or to solid plates, with the appropriate concentration as follows: kanamycin (50 μg mL^–1^) or chloramphenicol (35 μg mL^–1^), separately. The culture density was monitored by determining the optical density at a wavelength of 730 nm (OD_730_).

### *Synechocystis* Mutant Construction

To express mannitol in *Synechocystis* under control of a salt-inducible promoter, the mannitol cassette (*mtlD* and *m1p*) with a kanamycin resistance gene was amplified from ΔCS_M.^9^ Then this fragment, together with ∼1kb of upstream and downstream homologous regions of *ggpS*, was fused via overlap PCR. The fused fragment was sequenced to confirm the absence of mutations and then directly used to transform the ΔCS strain at the *ggpS* locus on the chromosome. Since *ggpS* was shown to be a salt-induced gene, its promoter was therefore assumed to be salt inducible as well and was used to control mannitol expression in this study.^37^

To knock out *pnp* from the parental strain ΔCS_M (OMP), the chloramphenicol resistance cassette was amplified from SAW011 ^31^ and fused with ∼1kb of upstream and downstream homologous regions of *pnp* via overlap PCR. This fused fragment was directly used for replacement of *pnp* in the OMP by the presence of chloramphenicol in BG-11 medium.

The method used for natural transformation in *Synechocystis* was essentially as described previously.^9^ In brief, *Synechocystis* cells in the exponential phase were collected from a shake flask, cultivated in an incubator until the OD_730_ reached ∼0.4, and concentrated 5 times by centrifugation at 5000 rpm for 5 min. Then, the two previously mentioned fused fragments were separately mixed in 200 μL corresponding concentrated cells at 10 μg mL,^1^ followed by 5 h of incubation in a red-light shaking incubator at 120 rpm. After incubation, mixed cells were spread onto a commercial membrane (Pall Corporation, USA) and this membrane was placed on a BG-11 solid plate with the corresponding antibiotics (kanamycin or chloramphenicol). Single colonies appeared on the membrane after around 10 days and the segregation status of mutants was confirmed by PCR. Once full segregation of the mannitol cassette was achieved by propagations in the presence of kanamycin, the mannitol cassette from *Synechocystis* was sequenced again to check for the absence of mutations. This mannitol-producer under control of the inducible promoter was named as ΔCS_IM. Complete removal of *pnp* from the OMP was not achieved, so we named the obtained partially segregated *pnp* deletion mutant as the OMP^#^1*. The information on primers used for mutant construction or sequencing is shown in Table S1.

### Adaptive Laboratory Evolution

Adaptive laboratory evolution (ALE) of ΔCS, ΔCS_IM, and ΔCS_M was carried out in 20 mL of liquid BG-11 medium with either 200 mM or increasing salt stress (starting at 200 mM) in a 100 mL shake flask under the previously mentioned cultivation conditions (Figure 1). Cells were diluted and inoculated into fresh medium at an OD_730_ of 0.05 from an OD_730_ of ∼2.0 to maintain the growth phase in three parallel cultures. Growth was monitored every 2 h during the day after dilution for a minimum 10 h, to reliably calculate the growth rate. The decision to increase salt concentration of the cultures in the increasing salt condition depended on the growth rate. The salt concentration was increased by 50 mM once the observed growth rate increased or stabilized. A representative population was sampled at the end of each ALE experiment and stored at −80 °C in a 20% glycerol solution. The frozen stock of ΔCS_M at the 164th generation was streaked on a BG-11 plate for single colony isolation. From the plate, 10 colonies were inoculated in liquid BG-11 medium with 200 mM salt stress, and of those, the three isolates with the highest extracellular mannitol productivity were stored at −80 °C in a 20% glycerol solution and labeled as EMP^#^1, EMP^#^2, and EMP^#^3 (Figure 2). In addition, three colonies of the parental strain of ΔCS_M were isolated after streaking on the BG-11 plate. These three were subsequently cultivated in liquid BG-11 with 200 mM salt to measure mannitol productivity and labeled as OMP^#^1, OMP^#^2, and OMP^#^3 (Figure 2).

### Measurements of Mannitol Concentrations

Extracellular mannitol concentration from both ΔCS_IM and ΔCS_M during ALE was determined in the supernatant collected from *Synechocystis* cultures when the OD_730_ reached roughly 2, using a D-Mannitol-L-Arabitol Assay Kit (Megazyme).^8^ Aliquots (typically 500 μL) of the culture were harvested by centrifugation (12,000 rpm, 1 min, 20 °C) to separate the culture supernatant and cells. Then, 100 μL of the supernatant samples was used for extracellular mannitol measurement based on the manufacture’s instruction. The extracellular mannitol production by OMP^#^1, OMP^#^2, OMP^#^3, EMP^#^1, EMP^#^2, and EMP^#^3 was determined when the OD_730_ reached 2 and after 14 days cultivations, using the procedure as described before. The total extracellular plus intracellular mannitol concentration was determined by measuring the mannitol concentration in the supernatant of lysed cell cultures. In detail, 200 μL was sampled either when the cultures’ OD_730_ reached 2 or after 14 days cultivation and subsequently mixed with 60% (w/v) 0.1 mm glass beads. The cells were subsequently disrupted in a Precellys 24 bead-beater (Bertin Technologies) by 20 s of beating at 6000 rpm, followed by 120 s on ice, which was repeated a total of ten times. Then, insoluble material and glass beads were removed by centrifugation at 10,000*g* and 4 °C for 15 min. The total mannitol concentration in the supernatant was determined with the D-Mannitol-L-Arabitol Assay Kit according to the manufacture’s protocol.

### Whole-Genome Resequencing

Genomic DNA of OMP^#^1, OMP^#^2, OMP^#^3, EMP^#^1, EMP^#^2, and EMP^#^3 was extracted as described in previous study^38^ and sequenced on the Illumina NextSeq 550 system in a pooled paired-ended library of 32Gb with a read size of 150 bp, according to the manufacturer’s protocol.

## Identification of Mutations

Obtained sequencing data (BioProject accession number: PRJNA1242250) was analyzed using a custom-made pipeline automated in Snakemake (see supplements). Trimmomatic^39^ was subsequently used to remove adapter sequences and low-quality reads from the data. Since *Synechocystis* is a polyploid organism,^22^ chromosomal mutations can take place without full segregation, i.e., without being present in every chromosomal copy. We therefore decided to use the breseq pipeline^21^ in polymorphism mode so we would also be able to detect the presence of nonsegregated mutations.

As reference, we used a modified version of the *Synechocystis* sp. PCC6803 GT-S reference assembly accessed via Genbank Assembly ID: GCF_000270265.1. We adjusted the reference sequence to contain the synthetic mutations present in the parental strain OMP (see supplements). This ensures breseq is able to detect mutations in areas not covered by the original reference.

We ran breseq on all samples independently and combined the resulting genome diff files with the COMPARE command from the gdtools utility. Including partially segregated mutations in the results dramatically increased the number of false positives. To identify mutations potentially relevant for explaining the increased mannitol productivity, we used Principal Component Analysis (PCA) and enrichment analysis. PCA looks for patterns in the data that discriminate between the samples in the data, and we selected those components that discriminate between the parental (OMP) and evolved (EMP) isolates. The 10 mutations with the largest absolute contribution to the selected PCs (measured through their loading) were considered as potentially relevant mutations.

With enrichment analysis, we treated the percentage of reads supporting the mutation as a measure of segregation. We calculated for both generations the average frequency of each mutation. In our experiment, we select for mutations that improve salt resistance through increased mannitol production, and we expect the presence of advantageous mutations to increase in the population at higher generations. We therefore marked the mutations with an increase in frequency as potentially relevant mutations.

So far, each mutation was treated independently in our analysis. However, ultimately, we wanted to associate genes to mannitol production. We noticed that some genes contained up to 80 different mutations at both increasing and decreasing frequencies. Since mutations decreasing in frequency cannot explain the increase in mannitol, we decided to ignore mutations occurring in genes that also contain one or more mutations at decreasing frequency.

Finally, we combined the results from PCA and enrichment analysis to determine which genes are most relevant in our study and that we will proceed to validate experimentally.

### Sanger Sequencing

To confirm the mutations in *rpoDI* and *pnp* in EMPs revealed by genomic resequencing, Sanger sequencing targeting these loci was subsequentially performed. Primers used for amplifying and sequencing *rpoDI* and *pnp* fragments are listed in Table S1.

### Determination of Salt Resistance

To test the salt tolerance of OMP^#^1, OMP^#^2, OMP^#^3, EMP^#^1, EMP^#^2, EMP^#^3, and OMP^#^1*, the cells at the log phase in *Synechocystis* cultures were first counted using a CASY 1 Model TTC cell counter (Schärfe System GmbH, Reutlingen, Germany) with a 60 μm diameter capillary and diluted to a total cell number of 12,500 cells μL^–1^. A 5 μL culture from each mutant, grown with 200 mM salt, was separately spotted on solid BG-11 plates with 0, 200, 250, 300, 350, 400, and 450 mM NaCl under the mentioned cultivation condition. Visible and green colonies appeared within 10 days.

## Supplementary Material



## Notes

Extracellular mannitol production and productivity of different mutants under varying salt concentrations; growth curves of OMP and EMP strains under varying salt concentrations; whole-genome sequencing analysis using breseq; PCR validation of partial *pnp* gene deletion; drop experiments of the OMP#1 and OMP#1* strains; and list of primers and strains used in this study (PDF)

## ‡

Zhejiang Huarui Biotechnology Co., Ltd. No.23, Zhongtian second Road, Huzhou City 313000, Zhejiang Province, China
