# Supplementary material for: Isolation of Synechocystis Mutants Overproducing Mannitol Directly from CO2 via Laboratory Evolution under Increasing Salt Concentration
Source: ACS Synth Biol. 2025 Sep 1;14(9):3557–67. doi: 10.1021/acssynbio.5c00344 (PMC12455643; doi:10.1021/acssynbio.5c00344)
Supplement: Supplementary file 1 [file sb5c00344_si_001.pdf]

Supplementary data

Isolation of *Synechocystis* mutants overproducing mannitol directly from CO_2 via laboratory evolution under increasing salt concentration

Wenyang Wu[1,a,b], Jente A. Jongbloets[1,a], Wei Du[1,a], Klaas J. Hellingwerf[a] and Filipe Branco dos Santos[a,*]

[a] Molecular Microbial Physiology Group, Swammerdam Institute for Life Sciences, Faculty of Sciences, University of Amsterdam, Science Park 904, Amsterdam 1098 XH, The Netherlands

[b] Present address: Zhejiang Huarui Biotechnology Co., Ltd. No.23, Zhongtian 2nd Road, Huzhou City 313000, Zhejiang Province, China

[1] These authors contributed equally to this work.

*Corresponding author
Filipe Branco dos Santos
f.brancodossantos@uva.nl

Figure S1. Extracellular mannitol production (A) and productivity (B) of ΔCS (*ggpS* and *sps* double knock out mutant), ΔCS_IM (mannitol cassette under salt-inducible P_{ggpS} promoter on the ΔCS background) and ΔCS_M (mannitol cassette under P_{trc1} promoter on the ΔCS background) during the process of adaptive laboratory evolution (ALE) under the constant 200 mM salt pressure.

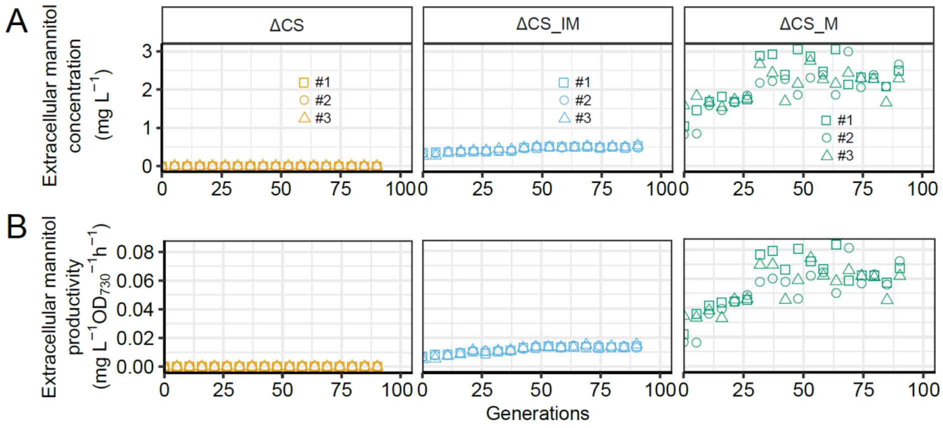

Figure S2. Extracellular mannitol production (A) and productivity (B) of ΔCS (*ggpS* and *sps* double knock out mutant), ΔCS_IM (mannitol cassette under salt-inducible P_{ggpS} promoter on the ΔCS background) and ΔCS_M (mannitol cassette under P_{trc1} promoter on the ΔCS background) during the process of ALE under the increasing salt pressure. The numbers in the plots indicate the salt concentrations (in mM) used during ALE.

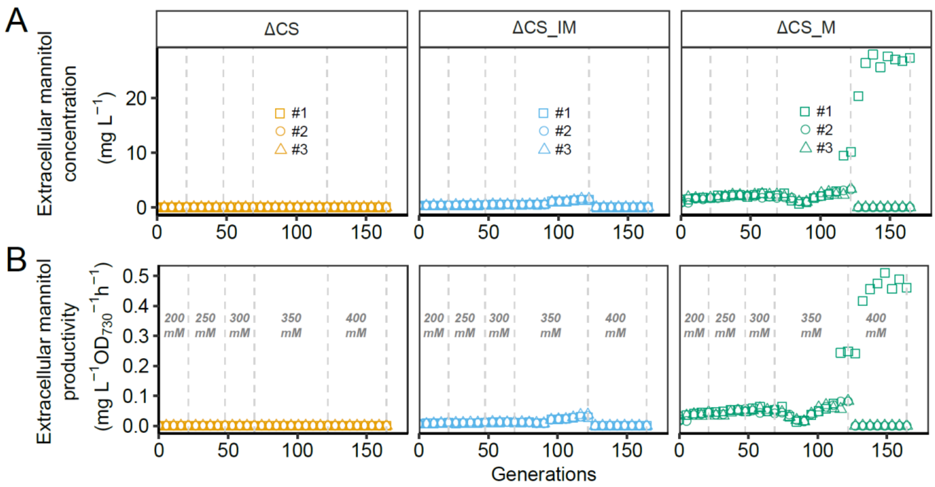

Figure S3. Growth curves of OMP and EMP under varying salt concentrations (0, 200 mM, 250 mM, 300 mM, 350 mM, 400 mM and 450 mM). Error bars represent the standard deviation from three biological replicates, each derived from a distinct isolate.

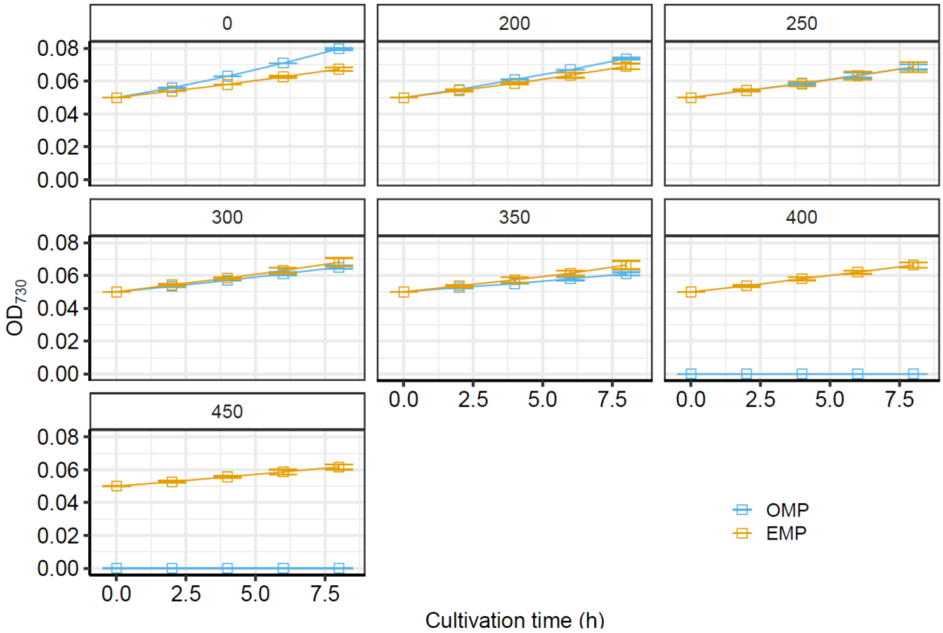

Figure S4. Comparison of OMP and EMP phenotypes under varying salt concentrations (0, 200, 250, 300, 350, 400, and 450 mM NaCl). (A) Total and extracellular mannitol production, and (B) mannitol productivity of OMP and EMP isolates at an OD_{730} of 2. Bars represent extracellular mannitol; empty squares indicate total mannitol. Error bars represent the standard deviation of three biological replicates.

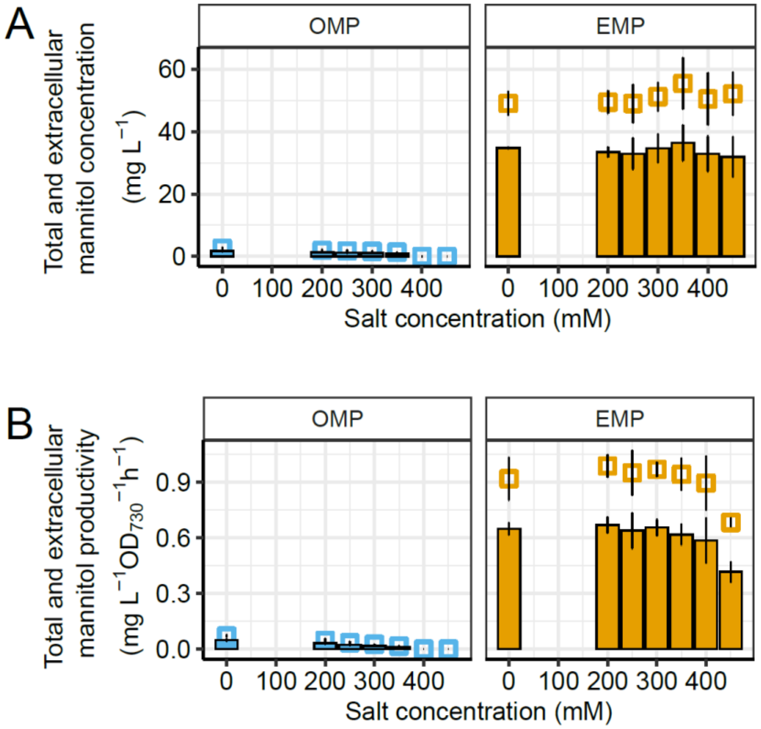

Figure S5. Growth curves of OMP and EMP isolates over 14 days of continuous cultivation under varying salt concentrations (0, 200, 250, 300, 350, 400, and 450 mM NaCl). Error bars represent the standard deviation of three biological replicates.

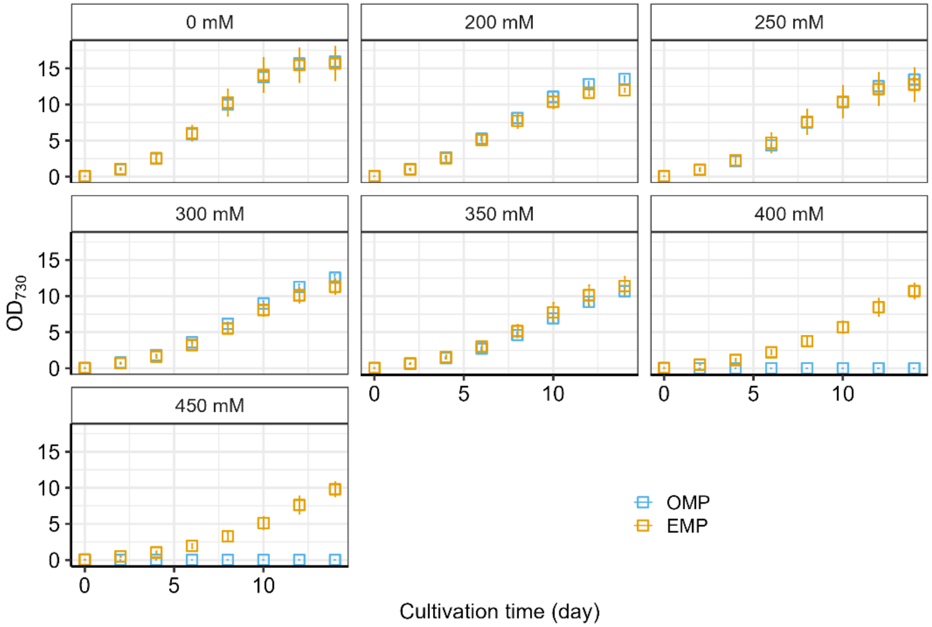

Figure S6. Total mannitol production by OMP and EMP isolates after 14 days of continuous cultivation under varying salt concentrations (0, 200, 250, 300, 350, 400, and 450 mM NaCl). Error bars represent the standard deviation of three biological replicates. 'ns' indicates no significant difference as determined by a two-tailed t-test.

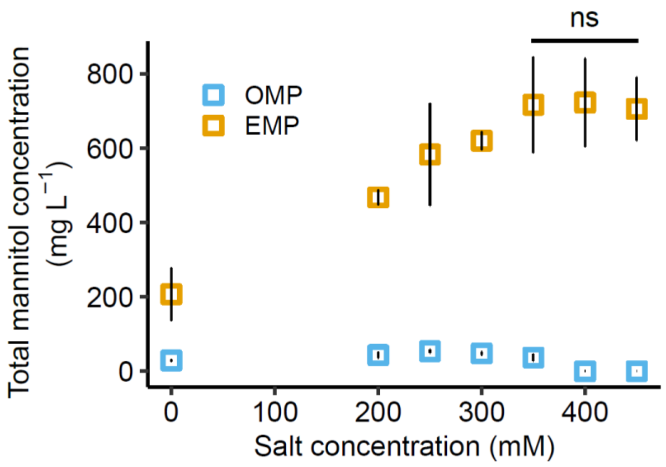

Figure S7. Results of Principal Component Analysis (PCA) on mutations detected in whole genome sequencing by breseq. (A) Variance explained by each Principal Component (PC), with the first two components explaining more than 75% of the variance in the dataset. (B) Clear separation of the different isolate generations is visible by PC 1 on x-axis and PC 2 on y-axis. Within the evolved isolates, EMP#2 stands out. (C, D) Heatmap showing the 10 most (in absolute sense) contributing mutations to PC 1 and 2 (respectively) and the corresponding percentage of reads supporting the mutation in each isolate (color). Mutations are labelled with the gene name in which they occur, the mutation ID and their loading in PC1. Isolates were clustered using hierarchical clustering.

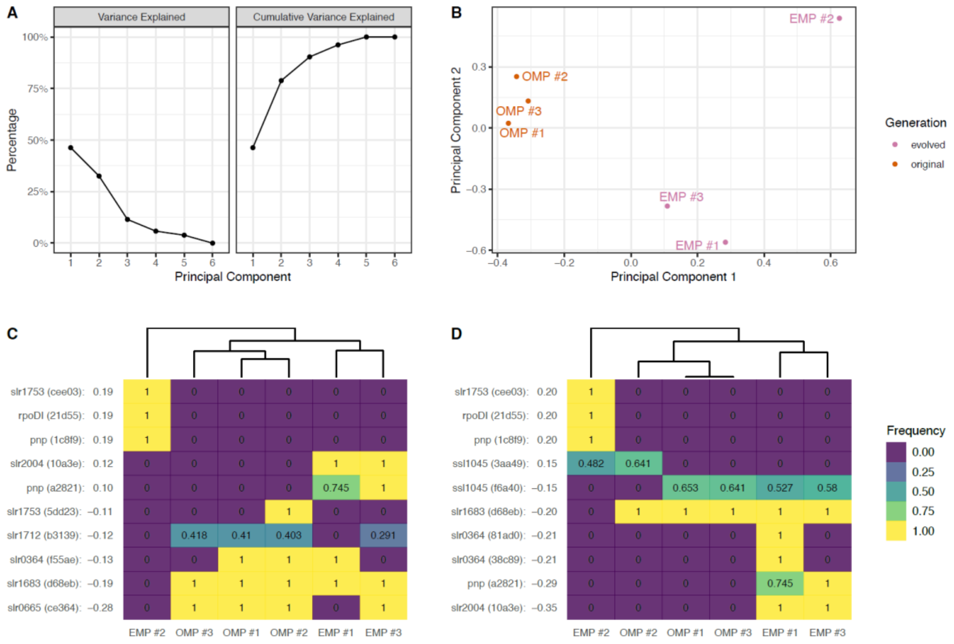

Figure S8. Results of Enrichment Analysis on mutations detected in whole genome sequencing by breseq. Heatmap shows the 10 most enriched mutations and the corresponding percentage of reads supporting the mutation in each isolate (color). Mutations are labelled with the gene name in which they occur, the mutation ID and the observed enrichment value. Isolates were clustered using hierarchical clustering.

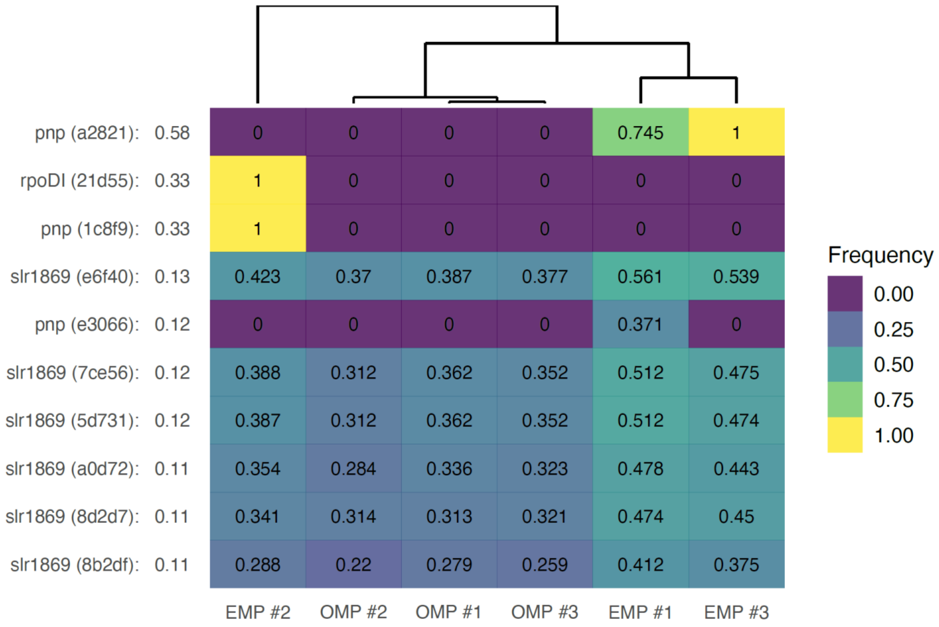

Figure S9. PCR validation of partial *pnp* gene deletion in the OMP#1* strain using primers Hom2PNP_F and Hom2PNP_R, which anneal to the ends of the homologous region. Successful replacement of *pnp* with the chloramphenicol resistance cassette yields a ~2.5 kb PCR product, whereas amplification from the wild-type chromosome produces a ~3 kb band.

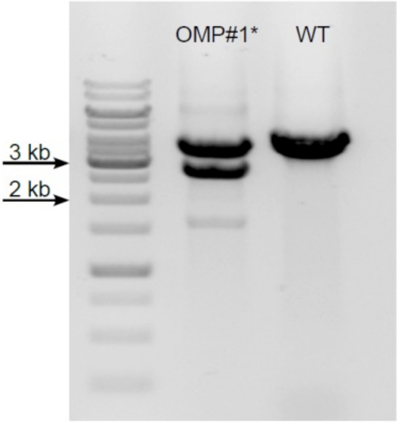

Figure S10. The effect of salt concentrations on the growth of OMP$^{\#}$1 and OMP$^{\#}$1*. The cell density was determined by casy counter and diluted as 12,500 cells μL^{-1}, 5 μl of OMP$^{\#}$1 and OMP$^{\#}$1* were transferred to solid BG-11plates with 0, 200 mM, 250 mM, 300 mM, 350 mM, 400mM and 450 mM salt. The plates were incubated for 10 days at 30ºC.

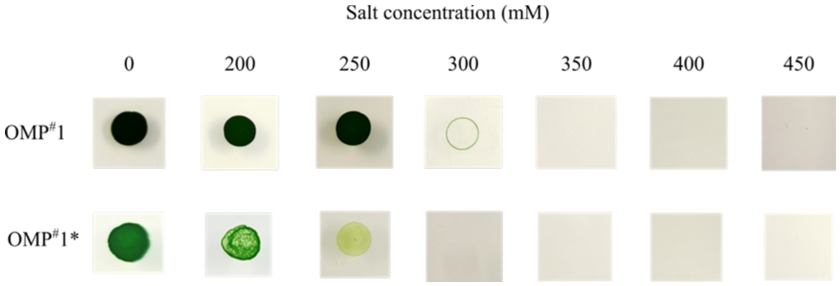

Figure S11. Total and extracellular mannitol production (A) and the productivity (B) of OMP[#]1 and OMP[#]1* when OD_{730} reached 2 under different salt concentration (0, 200 mM, 250 mM, 300 mM, 350 mM, 400 mM and 450 mM). Bars represent extracellular mannitol; empty squares indicate total mannitol. Error bars represent the standard deviation of three biological replicates.

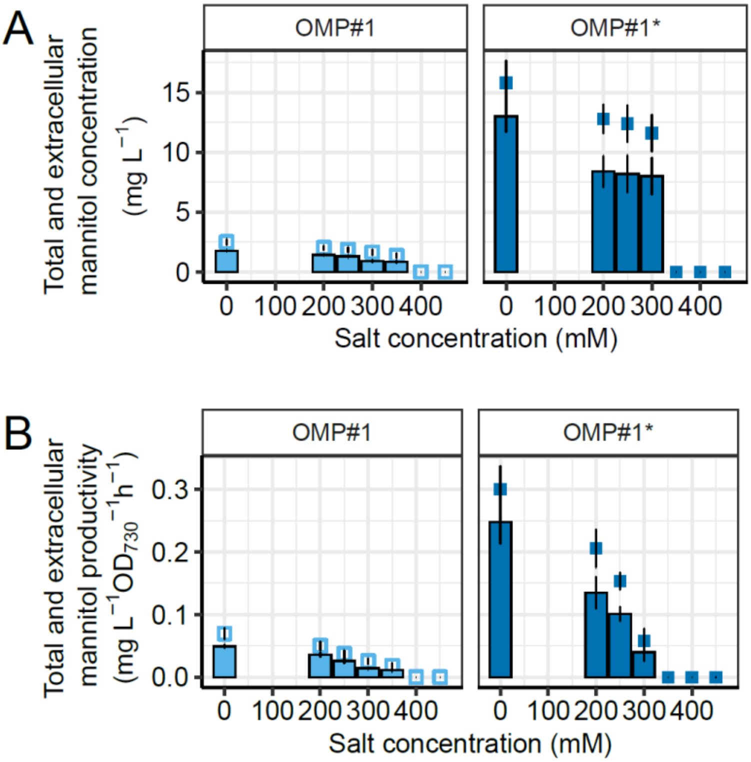

Figure S12. Total mannitol production of OMP#1 and OMP#1* after 14 days continuously cultivation under different salt concentration (0, 200 mM, 250 mM, 300 mM, 350 mM, 400 mM and 450 mM). Error bars represent the standard deviation of three biological replicates.

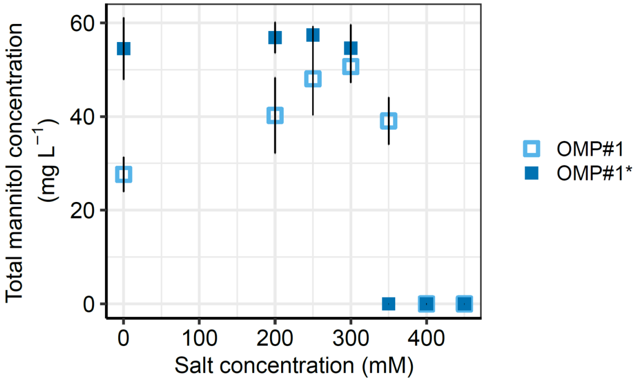

Table S1 Primers used for mutant construction and sequencing

Primer name	Sequence	Purpose
Seqmannitol_1	5'-ATACTGGCCATGCCATTACC-3'	Sequencing mannitol cassette for absence of mutation
Seqmannitol_2	5'-TATGCGAATAACCAACTCAC-3'	Sequencing mannitol cassette for absence of mutation
Seqmannitol_3	5'-TGTGCTAGGGCGGCCTGTGG-3'	Sequencing mannitol cassette for absence of mutation
Seqmannitol_4	5'-ATGCCGGGAATGTTGGGTAA-3'	Sequencing mannitol cassette for absence of mutation
Hom1GGPS_F	5'-TCCTTTCCCAACGAAACAAGCC-3'	Amplification of homologous region upstream the *ggpS* gene
Hom1GGPS_R	5'-CTGCAGTTTCTAGACCATATG-3'	Amplification of homologous region upstream the *ggpS* gene
Hom1mannitol_F	5'-CATATGGTCTAGAAACTGCAGATGAAAGCTTTGCACTTTGGC-3'	Amplification of mannitol cassette with kanamycin resistance cassette and fused with upstream of *ggpS*
Hom1mannitol_R	5'-CGCTGAGGTCTGCCTCGTGA-3'	Amplification of mannitol cassette with kanamycin resistance cassette and fused with downstream of *ggpS*
Hom2GGPS_F	5'-TCACGAGGCAGACCTCAGCGGCGATCGCCAATGCCAGTTG -3'	Amplification of homologous region downstream the *ggpS* gene and fused with kanamycin resistance cassette
Hom2GGPS_R	5'-TATCCACAAACGCTTCCACATA-3'	Amplification of homologous region downstream the *ggpS* gene
Hom1PNP_F	5'-ATCTTTACTAATTAGTGCTGC -3'	Amplification of homologous region upstream the *pnp* gene
Hom1PNP_R	5'-GAAGACGAAAGGGCATCGCGTTGTTAGTCAAAGACCATTA-3'	Amplification of homologous region upstream the *pnp* gene and fused with chloramphenicol resistance cassette
Hom1Cam_F	5'-TAATGGTCTTTGACTAACAACGCGATGCCCTTTCGTCTTC-3'	Amplification of chloramphenicol resistance cassette and fused with homologous region upstream the *pnp* gene
Hom1Cam_R	5'-ACATAAGGAAAACACGTTAA	Amplification of chloramphenicol resistance cassette and fused

	GATCGCGCGATGGGTCGAAT -3’	with homologous region downstream the *pnp* gene
Hom2PNP_F	5’-ATTCGACCCATCGCGCGATCTTAACGTGTTTTCCTTATGT-3’	Amplification of homologous region downstream the *pnp* gene and fused with chloramphenicol resistance cassette
Hom2PNP_R	5’-CAACAATGTCAGGCGGCGTTT-3’	Amplification of chloramphenicol resistance cassette
Seqpnp1	5’-TGGTACGGGTTGTGTCGGAA-3’	Sequencing *pnp* for absence of mutation
Seqpnp2	5’-ATTTCATCCCCTTCCTTGAT-3’	Sequencing *pnp* for absence of mutation
Seqpnp3	5’-AGAGGCTCTGTTTGCAATGA-3’	Sequencing *pnp* for absence of mutation
Hom1rpoDI_F	5’-ATCAATAGACTGACCGAGCGC-3’	Amplification of *rpoDI* from SMP[#]2
Hom1rpoDI _R	5’-TCGACACCGGTAACTACAAA-3’	Amplification of *rpoDI* from SMP[#]2
SeqrpoDI1	5’-CCGATTCATATATTTTTTGG-3’	Sequencing *rpoDI* for absence of mutation
SeqrpoDI2	5’-CCAAACAACGGTTAGTAGGC-3’	Sequencing *rpoDI* for absence of mutation
SeqrpoDI3	5’-AGGGGACTTCATCGAAGCCGA-3’	Sequencing *rpoDI* for absence of mutation

Table S2. Strains used in this study

Strains	Description	Reference
ΔCS_M	Mannitol cassette under Ptrc1 promoter on the ΔCS background	[1]
ΔCS	*Synechocystis* sp. PCC6803 *ggpS* and *sps* double gene knock out mutant	[1]
ΔCS_IM	Mannitol cassette under native promoter of *ggpS* on the ΔCS background	In this work
OMP[#]1*	Partially replacement *pnp* by chloramphenicol on the OMP[#]1 background	In this work

References

(1) Wu, W.; Du, W.; Gallego, R. P.; Hellingwerf, K. J.; van der Woude, A. D.; Branco dos Santos, F. Using Osmotic Stress to Stabilize Mannitol Production in Synechocystis Sp. PCC6803. *Biotechnol Biofuels* 2020, *13* (1), 117. https://doi.org/10.1186/s13068-020-01755-3.
